# Improved genome sequence of *Geobacillus kaustophilus* GBlys, a lysogenic strain by lysogenic phage phiOH2

**DOI:** 10.1128/mra.00906-24

**Published:** 2024-12-17

**Authors:** Rihito Tanaka, Soki Imbe, Yasuhiro Fujino, Yasuaki Hiromasa, Kazuki Mori, Kosuke Tashiro, Katsumi Doi

**Affiliations:** 1 Microbial Genetic Division, Institute of Genetic Resources, Faculty of Agriculture, Kyushu University, Fukuoka, Japan; 2 Center for Promoting International Education and Research of Agriculture, Kyushu University, Fukuoka, Japan; 3 Molecular Gene Technics, Graduate School of Bioresource and Bioenvironmental Sciences, Kyushu University, Fukuoka, Japan; Queens College Department of Biology, Queens, New York, USA

**Keywords:** *Geobacillus kaustophilus*, thermophiles, prophage, lysogenic phage

## Abstract

*Geobacillus kaustophilus* GBlys is a lysogenic strain of the temperate phage phiOH2, which is 3,644,428 bp long, has a GC content of 52%, and contains 3,595 predicted protein-coding genes. Here, we report a resequenced GBlys genome obtained by deep sequencing with long and short reads.

## ANNOUNCEMENT

The thermophilic bacterium *Geobacillus kaustophilus* GBlys is a strain lysogenized by the temperate phage phiOH2 of the type strain of this species, *G. kaustophilus* NBRC 102445^T^ (1). The complete genome sequence of strain GBlys was analyzed and determined using Ion PGM and 454 GS FLX, published in 2013 (2) (NCBI Assembly accession no. NZ_BASG00000000.1). However, 216 contigs and the phiOH2 prophage regions were fragmented. We then resequenced and reconstructed the genome of GBlys using state-of-the-art platforms and bioinformatics tools to produce an error-free full-length genome sequence.

Strain GBlys was cultured overnight at 55°C with shaking (150  rpm) in 802 broth (FUJIFILM Wako Pure Chemical Corp, Osaka, Japan) and DNA was extracted and purified by modifying the Marmur’s method (e.g., increasing the number of times the protein was removed using TE saturated phenol) (3). Sequencing involves the combination of short and long reads. Short-read sequencing libraries were constructed using the NEBNext Ultra II DNA Library Prep Kit for Illumina and decoded using the MiSeq system (Illumina, San Diego, CA, USA). A total of 856,978 reads with 214,654,552 bases were decoded, with an average insert size of 587 bp and a spot length of 2 × 250 bp paired ends. Low-quality bases (Q scores <15) were trimmed, and short reads (<200 bp) were removed using fastp v0.22.0 (https://github.com/OpenGene/fastp) (4). Long-read sequence libraries were constructed using a Ligation Sequence Kit (SQL-LSK110), and sequencing was performed on a MinION (Oxford Nanopore Technologies, Oxford, UK) system using a Spot-ON Flow Cell R9. No shearing and size selection were performed prior to library preparation. In total, 114,377 reads with 900,492,525 bases were decoded using Guppy ver. 5.0.12. The raw reads were filtered (quality, ≥10; read length, ≥1,000 bases, headcrop = 100) using NanoFilt v.2.8.0 (5), resulted in an N50 value of 21,907 bp. The trimmed long- and short-read data were assembled using Canu v2.2 and polished with Medaka v1.7.2 and Pilon v.1.23 (6). The genome completeness was confirmed using BUSCO version 4.1.2 (7), and the genome sequence was annotated using DFAST v1.2.18 (8). Default parameters of all software were used except where otherwise noted.

The complete GBlys chromosome was one contig of length 3,644,428 bp in length (52.2% GC content) with a sequence depth of approximately 200× (MinION) and 60× (MiSeq). In total, 3,595 predicted protein-coding genes, 88 tRNAs, 27 rRNAs, and 3 CRISPRS regions were annotated.

The genomic structure revealed that phiOH2 is a common feature of the family *Myoviridae*. The present results are interesting because Marks and Rowland described that all phages belonging to this family living in hot springs have been reported to be lytic (9). At present, seven strains of *G. kaustophilus* are registered in the NCBI database; however, none of them have regions homologous to phiOH2. Furthermore, all ORFs of phiOH2 had sequence homology with some proteins predicted to be present in the genomes of *Geobacillus* or *Anoxybacillus* spp., and 14 ORFs had sequence homology with phage proteins that infect *Geobacillus* or *Bacillus* spp.

## Data Availability

The genome sequence and annotation data for strain GBlys were deposited in DDBJ/GenBank under BioProject number PRJDB16840, BioSample number SAMD00653739, DRA numbers DRR590963 and DRR590964 and accession number AP035887.
